# Anal cytological abnormalities and epidemiological correlates among men who have sex with men at risk for HIV-1 infection

**DOI:** 10.1186/1471-2407-12-476

**Published:** 2012-10-16

**Authors:** Maria Gabriella Donà, Maria Benevolo, Amina Vocaturo, Guido Palamara, Alessandra Latini, Amalia Giglio, Domenico Moretto, Francesca Rollo, Giampaolo Impara, Fabrizio Ensoli, Fulvia Pimpinelli, Aldo Di Carlo, Massimo Giuliani

**Affiliations:** 1 Sexually Transmitted Infections (STI) Unit, San Gallicano Dermatological Institute, Via Elio Chianesi 53, 00144, Rome, Italy; 2 Pathology Department, Regina Elena Cancer Institute, Via Elio Chianesi 53, 00144, Rome, Italy; 3 Microbiology and Clinical Pathology Department, San Gallicano Dermatological Institute, Via Elio Chianesi 53, 00144, Rome, Italy; 4 Scientific Direction, San Gallicano Dermatological Institute, Via Elio Chianesi 53, 00144, Rome, Italy

**Keywords:** Anal cytology, HPV infection, Men who have sex with men

## Abstract

**Background:**

The incidence of anal cancer, a Human Papillomavirus (HPV)-related neoplasia, has been increasing in recent decades, mainly in men who have sex with men (MSM). Cytological changes of the anal epithelium induced by HPV can be detected through an anal pap smear. This study aimed to evaluate the prevalence and epidemiological correlates of anal cytological abnormalities among relatively young MSM at risk for HIV-1 infection, to help clarify whether or not this population deserves further investigation to assess the presence of anal cancer precursor lesions.

**Methods:**

MSM were recruited among attendees of a large STI clinic for a HIV-1 screening program. Anal samples, collected with a Dracon swab in PreservCyt, were used both for liquid-based cytology and HPV testing by the Linear Array HPV Genotyping Test. Data regarding socio-demographic characteristics and sexual behavior were collected in face-to-face interviews.

**Results:**

A total of 346 MSM were recruited (median age 32 years). Overall, 72.5% of the individuals had an anal HPV infection, with 56.1% of them being infected by oncogenic HPV genotypes. Anal cytological abnormalities were found in 29.8% of the cases (16.7% ASC-US and 13.1% L-SIL). Presence of ASC-US+ was strongly associated with infection by any HPV type (OR=4.21, 95% CI: 1.97-9.23), and particularly by HPV 16 and/or 18 (OR=5.62, 95% CI: 2.33-13.81). A higher proportion of ASC-US+ was found in older MSM, in those with a higher number of lifetime partners and in those with a history of ano-genital warts. However, none of these variables or the others analyzed showed any significant association with abnormal cytological findings.

**Conclusions:**

The presence of anal cytological abnormalities in about one third of the recruited MSM and their strong association with HPV infection, in particular that caused by HPV 16 and/or 18, might provide a further complement to the data that now support the introduction of HPV vaccination among MSM to protect them from the development of HPV-associated diseases. Additional studies are needed to determine whether and how screening for anal cancer precursor lesions should be performed in younger MSM.

## Background

Anal cytology has been adopted in some clinical settings as a standard procedure in individuals at risk for anal cancer in order to detect its precursor lesions (high-grade anal intraepithelial neoplasia, AIN). In fact, anal cancer incidence, which is very low in the general population [1], has been greatly increasing in recent decades, particularly in certain populations [2]. Men having sex with men (MSM) are at increased risk for the development of this neoplasia. Among these subjects anal cancer incidence is similar to that of cervical cancer before the introduction of cervical cytology screening, and is twice as high in those that are HIV-1 infected [3,4]. In general, history of receptive anal intercourse is strongly associated with occurrence of anal cancer, so representing a main risk factor for Human Papillomavirus (HPV) infection [5]. A persistent infection by HPV is now considered the cause of anal cancer, as well as of cervical cancer. It can be assumed that 85% of anal cancer cases occurring every year worldwide is caused by this virus as a result of a sexually transmitted infection [6], although HPV detection rate in this neoplasia varies according to gender, HIV status and sexual behavior [2,7,8]. Some studies have revealed the presence of HPV in virtually all cases of anal cancer diagnosed in MSM [2,8], with HPV16 being the most prevalent genotype (70-80%) [9,10]. Based on a recent meta-analysis, more than 60% of HIV-uninfected MSM have detectable anal HPV infections, and almost 20% of them show anal canal cytological abnormalities [11].

To date, only the New York State Department of Health has released specific recommendations for anal cancer screening in high-risk populations [12]. However, despite the wide consensus on the need to screen HIV-infected MSM, it is unclear if anal cytology should be recommended for other populations of MSM, such as younger individuals.

We conducted a cross-sectional study to assess the prevalence of anal cytological abnormalities, evaluated by liquid-based cytology, and anal HPV infection in a population of relatively young MSM at risk for HIV-1 infection. In addition, we analyzed the possible association of abnormal cytology with HPV infection and a number of selected socio-demographic and behavioral factors. These data should provide more information concerning the burden of anal cytological abnormalities among HIV-uninfected MSM.

## Methods

### Participant recruitment and questionnaires

Participants were recruited at the San Gallicano Dermatological Institute of Rome, Italy, from August 2009 to November 2011. The study group included MSM already participating in a longitudinal screening for HIV-1 and other sexually transmitted infections (STI), called the COROH Project. Participants, ≥18-year-old, that had not been previously vaccinated against HPV, were considered eligible according to the following criteria: 1. at least one receptive/insertive anal intercourse with a man in the preceding 6 months; 2. a HIV-1 negative antibody test at enrollment; 3. absence of anal or genital warts at enrollment; 4. no previous diagnosis of HPV-associated ano-genital cancers.

All participants underwent an accurate inspection of external genitalia and perianus in order to exclude the presence of ano-genital warts. Data on medical history, socio-demographic factors and sexual behavior were collected in face-to face interviews, which were conducted by a trained psychologist during the pre-HIV-test counseling session.

The study was approved by the ethics committee of the San Gallicano Dermatological Institute (Prot. CE/564/11) and was performed in compliance with the Helsinki Declaration. Informed consent was obtained from all the participants.

### Anal sample collection and liquid-based cytology

Anal samples were collected as previously described [13]. Briefly, a sterile Dracon swab was inserted and rotated into the anal canal, and was then vigorously agitated in the liquid cytology medium PreservCyt (Hologic) to dislodge the epithelial cells. Cytological slides were obtained using a ThinPrep 2000 processor (Hologic) and the GYN protocol. Cytology was independently interpreted by two certified cytopathologists, who were blinded to the HPV test results. Cytological findings were classified following the Bethesda 2001 guidelines, also accepted for anal cytology [14]. The Bethesda system includes the following categories: NILM (negative for intraepithelial lesion or malignancy), ASC-US (atypical squamous cells of undetermined significance), L-SIL (low-grade squamous intraepithelial lesion), or H-SIL (high-grade squamous intraepithelial lesion). Samples with poor cellularity or massive presence of squamous anucleated cells were considered inadequate for the cytological interpretation.

### HPV detection and genotyping

Molecular diagnosis of anal HPV infection was performed as previously described [13]. Briefly, total nucleic acids were extracted from 250 μl of the PreservCyt sample and screened for HPV DNA utilizing the Linear Array® HPV Genotyping Test (Roche Diagnostics) according to the manufacturer’s instructions. Results were considered valid whenever the amplification of the β-globin control and/or at least one HPV hybridization band were observed. The risk associated with the different genotypes was defined according to the classification adopted in one of our previous studies [13].

### Data analysis

To evaluate the association between cytological outcomes and the selected variables, crude odds ratios (COR) and 95% confidence intervals (CI) were calculated by univariate analysis using the SPSS+ package (vers.17.0). ASC-US and L-SIL cases were combined for purposes of analysis, and referred to as ASC-US+.

## Results

### Study population

A total of 346 HIV-uninfected MSM, who were mostly Caucasian (97.1%), were enrolled. The characteristics of the study population are shown in Table 1. The median age was 32 years (IQR: 27-39) and the median age at first intercourse with a man was 19 years (IQR: 17-23). The median number of sexual partners lifetime and during the previous 6 months were 50 (IQR: 20-200) and 5 (IQR: 2-10), respectively. Most of the participants had only casual partners (62.1%) and engaged in both receptive and insertive anal sex (66.1%).

**Table 1:** Selected socio-demographic and behavioral characteristics of the study participants

**CHARACTERISTIC**	
Age, median (IQR), years (*n*=346)	32 (27-39)
Ethnicity (%) (*n*=346)	
Caucasian	97.1
Hispanic	2.6
Asiatic	0.3
Education (%) (*n*=250)	
high-school or less	52.4
university or more	47.6
Annual income^a^ (%) (*n*=251)	
low	48.2
medium	42.6
high	9.2
Age at first intercourse with a man, median (IQR), years (*n*=254)	19 (17-23)
N. lifetime male sex partners, median (IQR) (*n*=245)	50 (20-200)
N. male sex partners in previous 6 months^b^, median (IQR) (*n*=264)	5 (2-10)
Partnership in previous 6 months^b^ (%) (*n*=264)	
steady partner only	14.0
steady and casual partners	23.9
casual partners only	62.1
Anal sex practices in previous 6 months^b^ (%) (*n*=227)	
insertive only	25.1
receptive only	8.8
receptive and insertive	66.1
Condom use during receptive intercourse in previous 6 months^b^ (%) (*n*=170)	
always	77.6
more than half times	15.9
up to half times	2.9
never	3.5
STI history^c^ (%) (*n*=264)	
No	59.1
Yes^d^	40.9
Genital herpes	4.6
Syphilis	31.5
Gonorrhea (any site)	35.2
Ano-genital warts	46.3

^a^ Low: <12,000 €; medium: 12,000-24,000 €; high: > 24,000 €.

^b^ During the 6 months preceding the enrollment.

^c^ STI diagnosed more than 6 months prior to enrollment.

^d^ Proportions of MSM with a specific STI were calculated as a percentage of the individuals with an STI history only; total sum of the percentages of patients with an STI history exceeds 100% due to patients with more than one STI.

Abbreviations: IQR, interquartile range; STI, sexually transmitted infections.

### Anal HPV infection

The presence of anal HPV was evaluated for all the participants, and 251 of them (72.5%) were found to be positive for the viral infection (any HPV type). High-Risk (HR) HPV types were detected in 56.1% of the cases, while 16.5% of the individuals were infected only by Low-Risk (LR) HPV types (Table 2). A single infection (only one HPV type in a sample) was revealed in 35.1% of the HPV-positive individuals. HPV16 was the most prevalent HPV type (18.2%, data not shown).

**Table 2:** Anal HPV infection distribution and prevalence overall, by HPV oncogenic risk, and number of genotypes

**HPV infection**	**N=346**	
	**n**	**%**
any type	251	72.5
any High-risk type	194	56.1
Low-risk only	57	16.5
High-risk only	81	23.4
Low-risk and High-risk	113	32.7
HPV 16 and/or 18	81	23.4
No. HPV types		
1	88	35.1^a^
2	68	27.1^a^
3	44	17.5^a^
≥ 4 (up to 10)	51	20.3^a^

^a^calculated as a proportion of the HPV-positive individuals.

### Anal cytology

**Figure 1:**
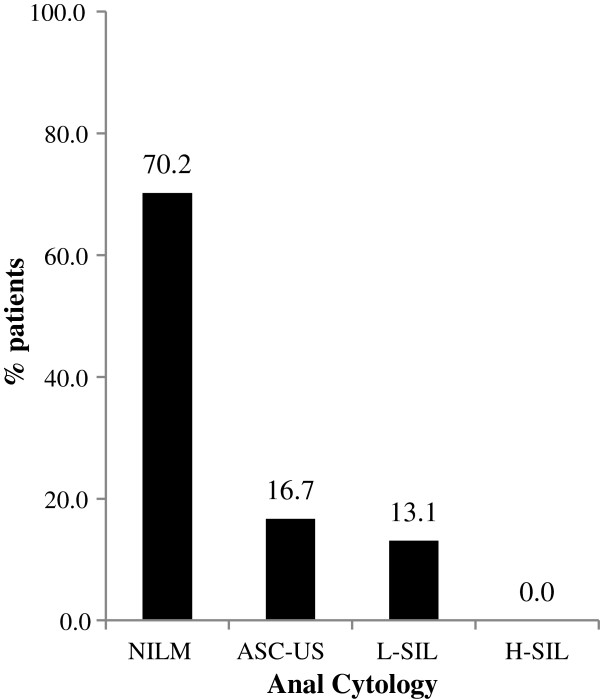
**Distribution of anal cytological abnormalities among 299 HIV-uninfected MSM.** Anal samples, collected with a Dracon swab and stored in PreservCyt, were used for liquid-based cytology to evaluate the presence of cytological abnormalities. These were classified following the Bethesda 2001 guidelines.

Among the 346 anal samples collected, 47 (13.6%) were considered inadequate for the cytological evaluation and not included in the following analysis. Notably, a valid HPV test result was obtained for all these cases, which displayed a prevalence of infection that was similar to the patients with a valid cytology report (data not shown). Among the 299 adequate samples, cytological investigation revealed normal cytology in 70.2% of cases, while ASC-US and L-SIL were reported for 16.7% and 13.1% of cases, respectively (Figure 1). None of the patients had a cytology report of H-SIL.

### Abnormal anal cytology associated factors

Possible associations between abnormal anal cytology, HPV infection, socio-demographic and behavioral characteristics were analyzed. We found a statistically significant association between abnormal anal cytology and HPV infection (any type), the prevalence of cytological abnormalities being 12.0% among HPV-negative individuals and 36.6% among HPV-positive MSM (COR=4.21, 95% CI: 1.97-9.23) (Table 3). All HPV-negative patients with cytological abnormalities had an ASC-US report. Compared to HPV-negative individuals, increased proportions of anal cytological abnormalities were evidenced both in patients infected by LR types only (12.0% vs. 38.5%, COR=4.56, 95% CI: 1.78-11.90) and in those with any HR type (12.0% vs. 36.0%, COR=4.10, 95% CI: 1.88-9.17). Importantly, MSM with HPV 16 and/or 18 anal infection showed the highest proportion of abnormal cytology (43.5%), which was more than three times higher than that found among HPV-negative participants (12.0%), COR=5.62, 95% CI: 2.33-13.81.

**Table 3:** Association between abnormal anal cytology and anal HPV infection in 299 HIV-uninfected MSM

	Abnormal anal cytology^*^n/N (%)	**COR (95% CI)**
**HPV infection**		
Neg	10/83 (12.0)	1.00
Any HPV	79/216 (36.6)	**4.21 (1.97-9.23)**
LR-HPV only	20/52 (38.5)	**4.56 (1.78-11.90)**
Any HR-HPV	59/164 (36.0)	**4.10 (1.88-9.17)**
HPV 16 and/or 18	30/69 (43.5)	**5.62 (2.33-13.81)**
HR-HPV other than 16 and/or 18	29/95 (30.5)	**3.21 (1.37-7.67)**
**Multiple HPV infection**		
No	21/77 (27.3)	1.00
Yes	58/139 (41.7)	1.91 (1.00-3.66)

Significant associations are highlighted in bold.

HR= High-Risk; LR= Low-Risk; COR= Crude Odds Ratio; CI= Confidence Interval.

*ASC-US+.

The prevalence of cytological abnormalities found among individuals infected by LR-HPV only was not significantly different from either that observed in MSM infected by any HR-HPV (38.5% vs. 36.0%, COR=0.90 95%, CI: 0.45-1.80) or that observed in MSM infected by HPV16 and/or 18 (38.5% vs. 43.5%, COR=1.23, 95% CI: 0.55-2.74).

The prevalence of cytological abnormalities was higher in patients with a multiple infection than in those with a single HPV type (41.7% vs. 27.3%), but this difference was only marginally statistically significant (COR=1.91, 95% CI: 1.00-3.66).

The positivity for HPV16 and/or 18 was higher in L-SIL than ASC-US cases (41.0% vs. 28.0%), while the positivity for any other HR-HPV was similar in the two cytology classes (33.3% and 32.0%, respectively, data not shown).

None of the selected socio-demographic and behavioral characteristics was associated with a significant increase in the prevalence of cytological abnormalities (Table 4). Although the proportion of ASC-US+ cases found in older MSM, in those with 20-49 lifetime sexual partners and in those with a history of ano-genital warts was higher than in the respective reference groups, in none of these cases was the increase statistically significant.

**Table 4:** Association between abnormal anal cytology and socio-demographic and behavioral characteristics of the study participants

**Variable**	Abnormal anal cytology* n/N (%)	**COR (95% CI)**
**Age (n=299)**		
18-24	13/52 (25.0)	1.00
25-29	21/69 (30.4)	1.31 (0.54-3.20)
30-34	17/55 (30.9)	1.34 (0.53-3.42)
35-39	18/58 (31.0)	1.35 (0.54-3.40)
40-44	9/35 (25.7)	1.04 (0.35-3.09)
≥45	11/30 (36.7)	1.74 (0.59-5.13)
**Education (n=221)**		
≤ High-school	33/115 (28.7)	1.00
>High-school	29/106 (27.3)	0.94 (0.50-1.76)
**Income**^**a**^**(n=221)**		
Low	33/110 (30.0)	1.00
Medium	26/93 (27.9)	0.91 (0.47-1.74)
High	5/18 (27.8)	0.90 (0.23-2.97)
**Age at first intercourse with a man (n=221)**		
≥21	25/80 (31.2)	1.00
16-20	32/106 (30.2)	0.95 (0.48-1.87)
≤15	7/35 (18.9)	0.55 (0.19-1.55)
**Lifetime number of male partners (n=213)**		
1-9	7/26 (26.9)	1.00
10-19	3/23 (13.0)	0.41 (0.06-2.15)
20-49	18/43 (41.9)	1.95 (0.61-6.45)
≥50	33/121 (27.3)	1.02 (0.36-2.96)
**Number of sexual partners in previous 6 months**^**b**^**(n=230)**		
≤5	36/126 (28.6)	1.00
6-30	26/88 (29.5)	1.05 (0.55-1.99)
>30	4/16 (25.0)	0.83 (0.18-2.99)
**Receptive anal sex in previous 6 months**^**b**^**(n=200)**		
No	12/46 (26.1)	1.00
Yes	41/154 (26.6)	1.03 (0.46-2.33)
**Condom use during receptive anal sex (n=155)**		
Always	35/119 (29.4)	1.00
Sometimes/never	7/36 (19.5)	0.58 (0.21-1.55)
**STI history**^**c**^**(n=232)**		
No	37/140 (26.4)	1.00
Other than ano-genital warts^d^	13/51 (25.5)	0.95 (0.43-2.10)
Ano-genital warts	16/41 (39.0)	1.78 (0.80-3.93)

^a^ Low: <12,000 €; medium: 12,000-24,000 €; high: > 24,000 €.

^b^ During the 6 months preceding the enrollment.

^c^STI diagnosed more than 6 months prior to enrollment.

^d^Genital herpes, syphilis, gonorrhea (any site).

COR= Crude Odds Ratio; CI= Confidence Interval.

*ASC-US+.

## Discussion

In the present study, we examined 346 MSM, with a high risk for HIV infection and other STI, as other studies have already shown [15,16], to assess the prevalence of anal cytological abnormalities and their association with anal HPV infection, socio-demographic and behavioral factors. Notably, this is one of the few studies conducted on relatively young MSM (median age 32 years), while most of the previous studies focused on older MSM [17,18]. Only a recent investigation was conducted on very young HIV-negative MSM, their median age being 22 years [19]. In addition, most of the research data on HPV and anal lesions have been collected in HIV-infected individuals, while this study focused on HIV-uninfected patients. It also represents, to our knowledge, the largest investigation conducted in Italy on this type of population.

This study was conducted on individuals with no visible HPV-related lesions in the anogenital area, which was assessed through the careful inspection of the external genitalia and perianal area. However, since no intra-anal examination was performed, neither through a digital rectal exam nor with high-resolution anoscopy (HRA), it cannot be excluded that a proportion of the individuals enrolled had intra-anal disease.

Our study showed that most of the subjects were infected by HPV in the anal canal, with an overall prevalence of 72.5%, which is a higher value compared to other cohorts of HIV-negative MSM [17,18]. The HPV testing methods used may account for differences between these studies. In addition, our results may also depend on the characteristics of the participants. In fact, as already mentioned above, they showed a high risk for HIV-1 infection and other STI [15,16]. HPV 16 was the most prevalent type in the present study, confirming that this genotype is the most frequently detected in the anal canal of both men and women [18,20-23].

The same samples used to assess anal HPV infection were also employed for liquid-based cytology. A proportion of these samples was not adequate for the cytological interpretation (13.6%), either because of scant cellularity or the massive presence of anucleated squamous cells. Due to the modality of the present study, conducted on STI Unit attendees who are not seen by scheduled appointments, it must be noted that it was not possible to ask the participants to adopt particular precautions prior to the collection of the anal sample, as otherwise indicated by the New York State Department of Health AIDS Institute [12] and by the Anal Neoplasia Clinic of the UCSF Comprehensive Cancer Center [24]. Therefore, patient behavior in the 24 hours preceding the exam, such as use of lubricants, might have compromised the quality of the specimen and might explain the observed proportion of inadequate samples.

Notably, a valid HPV test result was obtained for all samples, independently of the adequacy for the morphological evaluation, a fact that has also been observed in other studies [25]. This finding suggests that, although some samples lacked a sufficient number of nucleated cells to allow the cytological interpretation, the material was enough to obtain a valid HPV test result. This is probably due to the high sensitivity of PCR-based methods, which are able to detect virtually one copy of DNA.

Among the participants with a valid cytology report, we found abnormal anal cytology (ASC-US+) in 29.8% of the cases. This finding is consistent with results of previous studies conducted on HIV-uninfected MSM that report a prevalence of anal cytological abnormalities between 15 and 30% [26,27]. Notably, no H-SIL cases were found in the present study. This finding is probably due to the relatively young age of the participants, although a previous study conducted on a cohort of 262 HIV-negative MSM with a mean age of 45 years also failed to evidence any H-SIL cases at baseline [27]. Even though only anal H-SIL is considered the real precursor of anal cancer, it is worth noting that L-SIL may be clinically important. Previous studies have shown that about 40% of HIV-negative homo/bisexual men with L-SIL at baseline progressed to H-SIL within 2-4 years [26,27]. A diagnosis of ASC-US may also hide SILs, a fact also evidenced in earlier studies [28,29]. These data underline that all patients with a cytological report of ASC-US or worse need a follow-up or accurate diagnostic investigations, which may include high-resolution anoscopy and biopsy [30]. In fact, it is worth noting that one third of ASC-US and L-SIL reports may be associated with high-grade AIN diagnosed on a biopsy [31]. In addition, a recent study evidenced AIN2+ in 20% of patients with a negative cytology [32].

Importantly, infection by any HPV type was associated with a 4-fold increased risk of having abnormal anal cytology. MSM with a concurrent infection by LR-HPV and HR-HPV types had an increased prevalence of ASC-US+ compared to HPV-negative patients, but the highest rate of cytological abnormalities was found in patients with an infection by HPV16 and/or 18, which showed the strongest association with abnormal anal cytology (OR=5.62). Notably, previous studies have shown that infections with HPV16 or 18 are significant risk factors for the development of and progression to high-grade AIN (AIN-2, 3) [33,34].

We found a borderline correlation between abnormal anal cytology and HPV multiple infection. A previous study showed that a multiple infection was present with comparable frequency in men with normal cytology and those with ASC-US/L-SIL [35]. However, other reports have demonstrated an association between multiple HPV types and the presence of anal lesions [29,36]. Therefore, the possible role of multiple infections in the development of anal dysplasia remains unclear.

No association of abnormal cytology and age was found, a fact already observed in other studies that evidenced a similar prevalence of anal cytological abnormalities in all age groups [37]. We previously showed that HIV-negative MSM with a higher number of lifetime and recent sexual partners and having receptive anal sex were at increased risk of HPV infection [13]. Yet, none of these factors was significantly associated with abnormal anal cytology in the present study. It must be noted that receptive anal intercourse was indicated as a factor associated with anal cytological abnormalities in other studies [38]. However, it has been previously shown that anal HPV infection is not exclusively present in men reporting receptive sex [13,39,40], because the anal infection can also be acquired through other sexual practices. Therefore, it is not surprising to find a similar prevalence of abnormal anal cytology among MSM having and not having receptive intercourse. In addition, data collected for the present study referred to sexual behavior in the six months preceding the enrollment, thus it cannot be excluded that MSM not reporting receptive sex at the time of the interview had previously engaged in this practice. Finally, it is possible that our ability to identify statistically significant associations for some of the variables analyzed may have been limited by the small number of patients included in each group after stratification.

## Conclusions

Our data show that about one third of the relatively young HIV-uninfected MSM enrolled had anal cytological abnormalities. In addition, more than half of the whole population was infected by HR-HPV genotypes. Abnormal cytological findings were not significantly associated with any of the socio-demographic and behavioral factors analyzed. Conversely, a strong association with HPV infection, particularly with HPV 16 and/or 18, was evidenced. Our findings might provide a further complement to the large body of data that now support the introduction of HPV vaccination among MSM, who might greatly benefit from the prevention of HPV16/18 infection and related anal lesions.

All patients included in this study are currently in follow-up in order to monitor the possible development of anal dysplasia in cytologically negative participants and to evaluate the actual presence of AIN in individuals with abnormal anal cytology.

## Abbreviations

HPV: Human papillomavirus; MSM: Men having sex with men; HIV: Human immunodeficiency virus; STI: Sexually transmitted infection; SIL: Squamous intraepithelial lesion; NILM: Negative for intraepithelial lesion or malignancy; ASC-US: Atypical squamous cells of undetermined significance; L-SIL: Low-grade squamous intraepithelial lesion; H-SIL: High-grade squamous intraepithelial lesion; COR: Crude odds ratio; CI: Confidence interval; AIN: Anal intraepithelial neoplasia; HRA: High-resolution anoscopy.

## Competing interests

The authors declare that they have no competing interests.

## Authors' contributions

MGD carried out the molecular assays, participated in the design of the study and drafted the manuscript; MB and AV performed the cytological evaluation of the anal samples, the critical review of the results and the critical revision of the manuscript; FR prepared the cytological slides and managed the patient database; GP participated in conceiving the study and enrolled the patients; AL and GI enrolled the patients and performed the clinical examinations; AdC and FE participated in the study coordination; FP supervised the laboratory procedures; AG and DM collected and managed the anal samples; MG conceived and coordinated the study, performed the statistical analyses and helped draft the manuscript. All authors read and approved the final manuscript.

## Pre-publication history

The pre-publication history for this paper can be accessed here:

http://www.biomedcentral.com/1471-2407/12/476/prepub
